# Fungal Cell Wall Proteins and Signaling Pathways Form a Cytoprotective Network to Combat Stresses

**DOI:** 10.3390/jof7090739

**Published:** 2021-09-08

**Authors:** Chibuike Ibe, Carol A. Munro

**Affiliations:** 1 Department of Microbiology, Faculty of Biological Sciences, Abia State University, Uturu 441107, Nigeria; 2 Aberdeen Fungal Group, Institute of Medical Sciences, School of Medicine, Medical Sciences and Nutrition, University of Aberdeen, Aberdeen AB24 3FX, UK; c.a.munro@abdn.ac.uk

**Keywords:** fungi, cell wall, cell wall proteins, signaling pathways, stress tolerance

## Abstract

*Candida* species are part of the normal flora of humans, but once the immune system of the host is impaired and they escape from commensal niches, they shift from commensal to pathogen causing candidiasis. *Candida albicans* remains the primary cause of candidiasis, accounting for about 60% of the global candidiasis burden. The cell wall of *C. albicans* and related fungal pathogens forms the interface with the host, gives fungal cells their shape, and also provides protection against stresses. The cell wall is a dynamic organelle with great adaptive flexibility that allows remodeling, morphogenesis, and changes in its components in response to the environment. It is mainly composed of the inner polysaccharide rich layer (chitin, and β-glucan) and the outer protein coat (mannoproteins). The highly glycosylated protein coat mediates interactions between *C. albicans* cells and their environment, including reprograming of wall architecture in response to several conditions, such as carbon source, pH, high temperature, and morphogenesis. The mannoproteins are also associated with *C. albicans* adherence, drug resistance, and virulence. Vitally, the mannoproteins contribute to cell wall construction and especially cell wall remodeling when cells encounter physical and chemical stresses. This review describes the interconnected cell wall integrity (CWI) and stress-activated pathways (e.g., Hog1, Cek1, and Mkc1 mediated pathways) that regulates cell wall remodeling and the expression of some of the mannoproteins in *C. albicans* and other species. The mannoproteins of the surface coat is of great importance to pathogen survival, growth, and virulence, thus understanding their structure and function as well as regulatory mechanisms can pave the way for better management of candidiasis.

## 1. Introduction

*Candida albicans* is abundantly found in mammals. It often resides on the skin and mucosal layers of individuals as part of their normal flora. *C. albicans* causes a range of infections from superficial to life-threatening and systemic, dependent upon the host’s immune system [1] *C. albicans* uses an arsenal of pathogenic mechanisms to subdue or evade host immune responses [2,3]. The mannosylated surface protein coat is covalently linked to the skeletal cell wall polysaccharides and plays a vital role in mediating *C. albicans* interaction with the host. The proteins are not only important in maintaining cell wall integrity, masking the polysaccharide rich layer, therefore preventing recognition by dectin-1, but also contribute to virulence of this pathogen in many ways. They mediate adherence to host cells and indwelling medical devices, enable invasion of epithelial cells, facilitate biofilm formation, protect *C. albicans* against immune attack, coordinate communication between host cells and *C. albicans*, and are important in nutrient scavenging including zinc and iron [3]. Given the important roles of the cell surface proteins at every stage of *C. albicans* infection process, research has been focused on expanding our understanding of their biology and structure as well as their function in the cell wall [4]. This area is, however, rapidly expanding as the cell surface proteins have the potential to be a unique drug/vaccine target [5,6,7]. Proteomics analysis of purified cell wall material has shown that the walls contain about 20 different types of covalently bound cell wall proteins (CWPs) at any time and the protein profiles can change dramatically depending on the growth conditions [8]. In addition, the presence of particular cell surface proteins morphologically depends and correlates with either *C. albicans* yeast or hyphal form [8]. The aim of this review is to discuss the characteristics and functions of covalently bound CWPs, and how they are important for fitness and virulence, and enable the fungus to cope with host infection-induced stress conditions. The review will also discuss the regulatory mechanisms that control expression of cell wall-related genes and relate what is known in *C. albicans* and other *Candida* species.

## 2. Function of Cell Wall Proteins

Based on the existing model of the cell wall, it is made up of an inner polysaccharide rich layer and the outer protein coat [9,10,11]. A 3-D nanoscalar model of the *C. albicans* cell wall has been developed to probe accurate thickness and structure of the cell wall [4,10]. The investigators used an optimized 3-D electron tomogram and computer vision technique to make accurate measurements of cell wall thickness [4]. The scalar model developed gave a more refined prediction of the thickness of each cell wall layer and the precise structure of some of the wall components [4]. The inner layer of the cell wall is composed mainly of β-glucans (β-1,3-glucan and β-1,6-glucans), chitin microfibrils, and a small amount of mannosylated proteins is distributed throughout the inner layer [4]. Chitin (β-1,4-*N*-acetyl glucosamine) and β-glucans (β-1,3-glucan) are the main structural polysaccharides of the cell wall [12]. β-1,3-glucan forms a three-dimensional network comparable to a flexible wire spring, which explains the elastic nature of the cell wall and provides the platform for the attachment of β-1,6-glucan, CWPs, and chitin [13]. Chitin is covalently cross-linked to the β-1,3-glucan network and contributes to the rigidity and physical strength of the fungal cell wall [12]. The outer coat is made up of glycan fibrils post-translationally attached to CWPs that are vertically arranged perpendicular to the inner layer [4].

The outer coat of mannoproteins determines cell wall permeability and surface charge [9]. Restriction of cell wall permeability is due to the densely packed CWPs, the presence of bulky protein sidechains, and the formation of disulfide bridges between CWPs [12,14,15]. This feature protects the structural polysaccharides against enzyme degradation and dectin-1 receptor recognition [15,16]. The use of genomic and proteomic techniques has advanced our knowledge of the nature and abundance of these surface proteins. CWPs have a unique structure, they generally contain: an N terminus with a secretory motif and a C terminus [17]. They bear serine/threonine-*O*-manno-oligosaccharide and/or asparagine-*N*-glycan and may contain internal repeats and/or a glycosylphosphatidylinositol, GPI anchor attachment sequences [18,19]. The most abundant cell proteins are the GPI-modified proteins, which receive a GPI anchor during their passage through the secretory pathway [20,21,22,23,24] and constitute about 88% of the total wall mannosylated protein classes [25] (Table 1). The second class of CWPs are those with internal repeats, PIR-CWPs [18,26] (Table 1).

A 3-D electron tomogram was used to determine the structure of the outer coat of mannosylated proteins. The scalar architectural model of mannosylated proteins gave a more precise detail of their structure, location and molecular size including measurements of their length and branching [4,71]. The cell wall through the outer proteins mediates host pathogen-interaction. The scalar architectural model may be useful in investigating the structure–function relationships that support the fungal infection strategy [4,10].

CWPs have both enzymatic and structural functions and their population may differ in their abundances depending on environmental conditions, developmental stage and phase of the cell cycle [9]. During cell wall synthesis, the cell wall polysaccharides, chitin and β-1,3-glucan are synthesized by enzymes localized in the plasma membrane and are extruded out to the cell exterior and are then acted upon by wall-localized cell wall remodeling enzymes [17]. CWPs modify these cell wall polysaccharides and cross-link them, thus maintaining cell wall integrity [9]. The cross-linking between cell wall macromolecules extruded into the wall space is catalyzed by carbohydrate active cell wall remodeling enzymes, hydrolases, transferases, and transglycosidases that are located in the cell wall space [17,72]. Some of these enzymes include β-1,3-glucanosyltransferases, e.g., Phr family (see Table 1), which are a Gas-like family, Bgl2 (GH17), and Crh family representing chitin-glucanosyltransferases, these are cell wall-localized GPI anchored proteins [17,50,51,73,74,75,76]. *C. albicans phr1*Δ/Δ and *phr2*Δ/Δ mutants showed hypersensitivity to cell wall stressors such as Calcofluor white, CFW [45]. In *C. albicans*, synthetically lethal GPI-anchored proteins such as Dfg5 and Dcw1 (glycoside hydrolase (GH) family 76) are required for the incorporation of mannosylated proteins into the cell wall [55].

Structural surface proteins with no enzymatic activities such as flocculins (e.g., Flo1, Pga24), agglutinins (e.g., Als1, Rbt1, Hwp1), or β-1,3-glucan cross connectors (e.g., Pir1) that can form a scaffold for the attachment of other wall components, are important for cell:cell interactions and wall integrity [27,30,31,33,77,78,79,80,81]. Ssr1, a structural protein has been shown to contribute to normal cell wall architecture [82]. Pga59 is thought to be associated with the formation of a coat around the cell wall that can restrict cell wall permeability [64]. CWPs are also associated with virulence, biofilm formation, and coping with stress in fungi [33,36,61,67,68,83,84,85]. The following are some examples. The *ALS* gene family encodes eight GPI modified cell surface glycoproteins with peptide binding ability Ig-fold domain at the N terminus [86]. The Ig-fold mediates adhesion to fibronectin and other specific host proteins [87], and cell to cell aggregation through Als to Als interaction (Nobile et al., 2008). Heterologous expression of Als proteins in a nonadherent *S. cerevisiae* strain demonstrated that the Als proteins promote attachment to different surfaces (Nobile et al., 2008). The *als3*Δ/Δ mutant has reduced virulence in a murine model of oropharyngeal candidiasis [31]. Hwp1 N terminus contains a secretory signal sequence rich in proline and glutamine that is cross-linked by host transglutaminase to epithelial cells enabling the attachment of *C. albicans* to human buccal epithelial cells [39,88,89]. *C. albicans hwp1*∆ mutant has reduced ability to bind to human buccal epithelial cells and has poor translocation from the mouse intestine into the bloodstream, demonstrating a role for Hwp1 in disseminated candidiasis [31]. Attachment to host cells by *C. albicans* can also be due to morphology-independent covalently bound wall proteins, Hyr1, Ecm33, Iff4, and Eap1, covalently bound wall proteins, Phr1, and cell-surface associated proteases, Sap9 and Sap10 [90,91].

*C. albicans* can use endocytosis (through interaction of Als3 with host cadherins) or active penetration to invade the host cell [31]. After *C. albicans* adhesion to the host cell surface and hyphal germination and growth, there are hyphal-induced hydrolytic enzymes that facilitate host cell degradation. They particularly aid active penetration into host cells and damage tissues [92].

*C. albicans* expresses ten secreted aspartyl proteinase (Sap) isoenzymes. Each mature Sap protein contains two aspartic acid residues conserved within the active site and a conserved cysteine residue that plays a structural role. Sap1–8 are secreted and released to the environment, whereas Sap9 and Sap10 are cell surface bound [63,93,94]. Sap proteins have been linked to the ability of *C. albicans* to adhere to and damage host tissue as well as the ability to evade the host immune response [95]. Sap9 and Sap10 have proteolytic activity on non-basic, basic, and dibasic peptides and have targeted Cht2, Ecm33, Pga4, Ywp1, Als2, Rhd3, Rbt5, and glucan cross-linked protein, Pir1 as substrates. *C. albicans sap9*∆/∆ and *sap10*∆/∆ mutants demonstrated reduction in cell wall-associated Cht2 activity suggesting a direct influence of Sap9 and Sap10 activity on Cht2 function and a role in maintaining cell wall integrity [96].

During *C. albicans* infection, Als family, and Eap1 adhesin, are involved in the development of biofilms, an important virulent attribute. The fungus forms biofilms when it encounters solid surfaces such as indwelling medical devices, where fungal cells are encapsulated in a dense extracellular matrix, which sequesters antifungal drugs promoting drug resistance and persistence in the host [97,98]. Biofilm formation by *C. albicans* has been shown to be under the positive regulatory control of the transcription factor, Bcr1. Bcr1 regulates the expression of Als1, Als3, and Hwp1 [34,35,36]. These proteins in addition to Als2 are associated with various stages of biofilm formation in *C. albicans* [28,99].

The cell wall during growth requires continuous remodeling of its macromolecular network [17]. During cell wall stress, a fungus can also rapidly remodel its wall and adapt the composition of the new cell wall [52,73,100]. For example, in exposure to cell damaging antifungal drugs, *C. albicans* triggers cell wall rescue mechanisms that influence the expression of wall biosynthetic genes and CWPs [4,60,101]. Rescuing the cell wall requires stress signals that activate the cell wall integrity (CWI) pathways. Cell surface proteins that function as mechanosensors primarily are responsible for activating these CWI pathways. These proteins (Wsc1-3 and Mtl1) act like linear nanosprings that detect and transmit cell wall damage or stress [102,103,104,105] to the downstream receptors in the signaling pathways. The sensors have an overall similar structure in that they contain in their sequences: short C terminal cytoplasmic domains, a single transmembrane domain, and a periplasmic ectodomain that is rich in Ser/Thr residues [106]. The Ser/Thr-rich regions are highly *O*-mannosylated, accounting for extension and stiffening of the proteins. Thus, these polypeptides have been proposed as mechanosensors that act as rigid probes of the extracellular matrix [106,107]. Functionally, signals are received and transmitted through the highly *O*-mannosylated extracellular domains and phosphatidylinositol (PI)-4,5-bisphosphate, which recruits the N terminal domains of the Rom1/2-guanine nucleotide exchange factors through the plasma membrane, the sensors stimulate nucleotide exchange on Rho1 [102,105]. The various effectors of Rho1 include β-1,3-glucan synthase, β-1,3-glucan synthase activity, and Pkc1-activated MAPK cascade [104].

In summary, CWPs have a wide range of diverse functions that contribute to virulence, to maintenance of wall structure to ensure cellular integrity remains intact, and to sensing and transmitting signals from the environment. Many CWPs have been functionally characterized and their amino acid sequences are known, but only a handful have had their structures fully elucidated. Structure has a functional implication and understanding CWP structure can increase our knowledge of their functions, including roles in cell wall biogenesis.

## 3. Fungal Cell Wall Remodeling and Signaling Pathways That Are Activated in Response to Stress

*C. albicans* has been shown to grow at a high concentration of caspofungin a phenomenon called paradoxical growth. Paradoxical growth, in *C. albicans* is associated with induced cell aggregation and an increase in cell volume and cell wall chitin content [108]. In *C. auris*, however, it only induced an increase in cell wall chitin content [108]. Genes encoding Fks1 and Fks2 harboring the single nucleotide polymorphisms hot spot regions have been identified in *C. auris* [108]. The Fks2 carries the F635Y mutation that confers intrinsic echinocandin resistance on *Candida glabrata* [108]. Interestingly, *C. auris* RNA-seq data showed that paradoxical growth activates genes encoding cell membrane proteins and GPI-modified proteins required for cell wall damage response, chitin synthase, and MAPKs such as Mkc1, and Hog1 involved in maintaining cell wall integrity [108]. Fungal pathogens activate a lot of pathways to successfully adapt to caspofugin stress.

**Figure 1:**
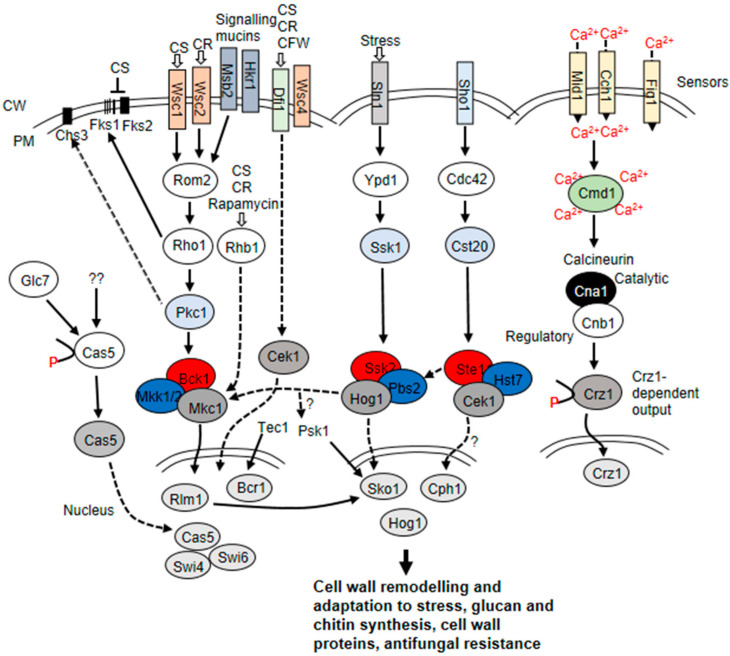
Signaling pathways that regulate cell wall remodeling of *S. cerevisiae* and *C. albicans*. The Hog1, Cek1, and Pkc MAP kinase cascades and the Ca^2+^/calcineurin signaling pathway control a number of cellular processes including cell wall synthesis and maintenance. Upstream membrane sensors of the MAP kinase cascades include Wsc family, Dfi1, Sho1, and Sln1, detect signals reporting weakened cell wall or alterations in the wall, and convey the signal to the downstream components of the pathway. The PKC pathway plays an important role in response to caspofungin and activates Rho1, a regulatory sub-unit of β-1,3-glucan synthase. Rhb1, an Rheb-related GTPase, activate the CWI MAP kinase Mkc1 in response to cell wall stress. An Rhb1 deletion mutant is hypersensitive to cell wall stress and to rapamycin [121]. Rho1 activates protein kinase C, which phosphorylates and activates Bck1 in the MAP kinase cascade. Bck1 in turn activates the MAP kinase kinases Mkk1/2, which then phosphorylate Mkc1, which may hypothetically target Rlm1 in *C. albicans*. Although the Mkc1–Rlm1 relationship has been shown in *S. cerevisiae*, there is no evidence in *C. albicans* that Rlm1 is downstream of the Pkc pathway. A number of transcription factors contribute to the echinocandin stress response including Cas5 and Bcr1 [54]. In *C. albicans*, Cas5 is activated through an unknown mechanism involving dephosphorylation by Glc7 phosphatase [122]. Cas5 interacts with Swi4 and Swi6 to activate Cas5-dependent gene transcription leading to the upregulation of genes involved in cell biogenesis/integrity and cellular metabolism [122]. Cas5 and Efg1 have been shown to interact in response to caspofungin stress. Efg1 regulates the transcriptional response to cell wall damage by caspofungin [123]. *C. albicans efg1*∆/∆ mutant is hypersensitive to caspofungin [123,124]. Cas5 and Efg1 coregulate the expression of caspofungin-inducible genes. Cek1 pathway impinges on cell wall regulation and has also been implicated in systemic candidiasis [119,125]. *C. albicans* Dfi1, a homologue of *S. cerevisiae* Mid2/Mtl1 is known to partly activate the MAP kinase Cek1 and confer tolerance to caspofungin, CR, and CFW [126]. A Dfi1 deletion mutant is severely affected in invasive filamentation and virulence in a murine infection model. Msb2 in cooperation with Sho1 is also thought to play a role in Cek1 activation [127]. It is predicted that the transcription factor, Cph1 a homologue of *Sc*Stel2, is downstream of the Cek1 mediated pathway [112,119,127]. Cph1 is associated with regulation of filamentation [127]. The Rlm1 and Bcr1 transcription factors control the expression of a number of cell wall-related genes [34,128] with Bcr1 playing a dominant role in the regulation of biofilm formation by controlling expression of several important adhesins. In *C. albicans*, the Rlm1 activation mechanism is unknown, but once localized in the nucleus, activated Rlm1 leads to the upregulation of genes involves in cell wall biogenesis/integrity, macromolecular localization, and organelle localization [129]. Putative Rlm1 binding motifs in the promoters of *CHS2* and *CHS8* contribute to their cell wall stress-activated regulation [10,130,131]. In *S. cerevisiae*, Pkc1 is involved in targeting Chs3 to the plasma membrane in response to heat shock [129,132]. Significant re-wiring of signaling pathways is evident in *C. albicans*, compared to the *S. cerevisiae* paradigm, for example, the role of the Sko1 transcription factor in response to caspofungin is independent of Hog1 MAP kinase, but involves the Psk1 PAK kinase [133] and Rlm1. In *C. albicans*, Sko1 regulates the expression of some genes involved in cell wall biogenesis and remodeling, and osmoadaptation [133]. Sko1 binding motif has been identified for regulating Sko1-dependent genes. Sko1 also binds to its motif to promote self-activation. The calcineurin pathway is activated by calcium that may enter the cells through membrane-localized channels Cch1 and Mid1 or a third minor channel Fig1. Alternatively, the pathway may be activated by calcium released from intracellular stores. Ca^2+^ binds to and activates calmodulin (Cmd1) that in turn activates the phosphatase calcineurin. The calcineurin is made up of two sub-units, Cna1 and Cnb1. Calcineurin dephosphorylates the transcription factor Crz1, which moves into the nucleus and induces expression of genes through binding to CDREs (calcium dependent response elements) within their promoter sequences. Two Crz1 DNA binding motifs have been identified in some genes regulated by Crz1. Adapted from [112,114,126]. CR = Congo red, CS = caspofungin, CFW calcofluor white, CWM = cell wall matrix, PM = plasma membrane.

Deletion of cell wall biosynthetic pathway genes in fungi often results in increased susceptibility of the cell wall to wall perturbing agents as well as alterations in chitin and β-1,3-glucan contents and linkages in the cell wall, synthesis of new wall proteins, and changes in the crosslinking to alternative wall polysaccharides [109,110]. Inhibition of β-1,3-glucan synthesis has been associated with altered crosslinking of chitin to β-1,6-glucan-GPI-modified proteins in the cell wall [109]. The amount of chitin→β-glucan←GPI-CWP complexes in the cell wall increased to 40% in wall defective mutants, indicating this is a repair mechanism protecting the cell wall from degrading enzymes and other stresses [109]. Most cell wall restructuring processes do not involve activation of the signaling pathways. For example, the carbon-source-induced alteration in osmotic tolerance in *C. albicans* was shown to be independent of the CWI pathways, but rather mediated by alterations in the architecture and biophysical properties of the cell wall [111]. However, during the cell wall response to most stressors, signals that indicate weaknesses in the wall are received by the surface sensors and transmitted leading to activation of the corresponding CWI pathways. In *Saccharomyces cerevisiae* and *C. albicans*, signaling pathways are activated in response to a wide range of stresses such as CFW, harsh temperatures, oxygen starvation, host immune response during infection and antifungal such as echinocandins, altered nutrient levels, and carbon source [109,112,113,114]. Cell wall stress response is mediated through the protein kinase C, PKC cell integrity mitogen-activated protein (MAP) kinase cascade, and its downstream transcription factors [112,114,115] (Figure 1). Other MAP kinase cascades, the high osmolarity glycerol response, HOG, and Candida ERK-like kinase, Cek1 mediated pathways, have also been shown to play a role in the cell wall reconstruction process [112,116,117] (Figure 1). MAP kinase defective *C. albicans* mutants display attenuated virulence in infection models showing that MAP kinase pathways are also important for virulence [118,119,120].

There is some redundancy in the regulatory networks responding to echinocandin-induced cell wall damage where more than one transcription factor controls overlapping sets of downstream target genes to control changes in the cell wall [54,122,133,134]. Three transcription factors, Cas5, Sko1, and Rlm1 have been implicated in echinocandin-induced cell wall damage signaling [54,133].

Cas5 has been shown to be involved in cell wall remodeling in *C. albicans* during cell growth, morphology, and virulence [54,122,135,136]. *C. albicans cas5*Δ/Δ mutants and including mutants with a missense mutation in Cas5 DNA-binding domain is hypersensitive to caspofungin and other cell wall stressors such as CFW [54,122]. A *cas5*Δ/Δ deletion mutant has also been shown to have attenuated virulence in both murine and invertebrate models of systemic candidiasis [135]. Genome-wide microarray studies showed that Cas5 regulates about 50% of the highly expressed caspofungin-inducible genes, including some cell wall integrity genes [54]. Studies using RNA polymerase II chromatin immunoprecipitation and sequencing analyses showed that the number of caspofungin-inducible genes is markedly higher and genes with cell wall-associated functions were markedly overrepresented [122]. Furthermore, Cas5 was found to regulate over 60% of caspofungin-inducible genes, including those involved in cell wall integrity [122].

Information on the upstream regulation of Cas5 is limited in *C. albicans*, but available data suggest that Cas5 is dephosphorylated by phosphatase Glc7 following caspofungin-induced cell wall damage [122]. The study further showed that upon dephosphorylation of Cas5, it is activated and interacts with Swi4 and Swi6 to activate the transcription of Cas5-dependent genes [122]. This leads to the upregulation of genes involved in cell wall synthesis/integrity and cell metabolism [122].

Cas5 together with Efg1 regulate the transcriptional response to cell wall damage by caspofungin [123]. Efg1 is a member of the APSES family of basic helix-loop-helix transcriptional regulators that is proposed to function downstream of the cAMP/protein kinase A (PKA) pathway to induce a hyphal transcription program [137,138]. Likewise, Efg1 is important for transcriptional responses to echinocandins and *C. albicans efg1*∆/∆ mutant is hypersensitive to caspofungin [124]. Efg1 also required for the induction of *CAS5* in response to cell wall damage by caspofungin [125]. Deletion of *EFG1* in a *cas5*∆/∆ mutant exacerbates caspofungin hypersensitivity and make caspofugin-resistant *C. albicans* sensitive again. The ectopical expression of *CAS5* could not salvage the growth defect of *C. albicans efg1*∆/∆ mutant treated with caspofungin [123]. Genome wide transcription profiling of *C. albicans cas5*∆/∆ and *efg1*∆/∆ mutants using RNA-Seq showed that Cas5 and Efg1 can coregulate the expression of caspofungin-inducible genes and can also independently regulate some genes [123]. Using yeast two-hybrid and in vivo immunoprecipitation, Cas5 and Efg1 were shown to interact and bind to the promoter of some caspofungin-inducible genes to coordinately activate their expression [123].

Efg1 has also been shown to regulate Czf1 expression [139,140]. Czf1, a *C. albicans* zinc finger cluster transcription factor, is required for white-opaque switching and filamentation [141]. Efg1 and Czf1 interact in a yeast two-hybrid experiment [140] and coordinate responses to farnesol during quorum sensing and white-opaque thermal dimorphism [142]. In the screen of a library of genetically activated forms of zinc cluster transcription factors, hyperactive Czf1 was found to have a cell wall associated function in *C. albicans* [143]. Hyperactive Czf1 drives the expression of many CWPs with cell wall associated functions that can induce a physical change in the cell wall architecture and rescue the hypersensitivity of different CWI partway deletion mutants to cell wall perturbing agents [143]. In addition, *C. albicans czf1*Δ/Δ mutant is hypersensitive to caspofungin [143].

Downstream of the Pkc pathway is the transcription factor, Rlm1. Rlm1 has been extensively studied in *S. cerevisiae* where it is the main transcriptional regulator of the Pkc CWI pathway [144,145]. However, our understanding of the function of the protein is limited in *C. albicans*. *C. albicans rlm1*Δ/Δ mutant is hypersensitive to CFW and Congo red [54] and analysis of mutant cell wall composition compared to wild type showed marked reduction in mannan composition and an increase in chitin levels [128]. This suggested that Rlm1 is involved in caspofungin induced CWI signaling. These characteristics of *rlm1*Δ/Δ in *C. albicans* have not been observed in *S. cerevisiae*, showing divergence of these orthologues [128]. In *C. glabrata,* which is more closely related to *S. cerevisiae* than *C. albicans*, *rlm1*Δ/Δ, *mkk1*Δ/Δ, and *bck1*Δ/Δ mutants are sensitive to caspofungin, but not to CFW or Congo red [146] and the full influence of this pathway on cell wall regulation is yet to be studied. Genome-wide microarray studies in *C. albicans* showed that Rlm1 only induced the expression of five genes under basal condition and only two of these genes were caspofungin-inducible [54]. Another genome-wide study demonstrated that Rlm1 regulated the expression of 773 genes under basal conditions [128] and some of the highly upregulated genes have cell wall associated function. These data suggest that Rlm1 may have a more general regulatory role in controlling cell wall associated gene during non-stressed physiological activities. Genome-wide ChIP Seq data revealed that Rlm1-target genes encode proteins that have cell wall-associated function [134]. Rlm1 bound to the upstream intergenic regions of 25 genes and 18 of the genes were highly caspofungin-inducible [134]. Furthermore, a *rlm1*Δ/Δ mutant attenuated virulence in a murine model of systemic candidiasis [128].

Orthologues of Pkc pathway are conserved in *C. albicans*; however, it is not known if the Mkc1 directly or indirectly activates Rlm1. Genomic, biochemical, and cellular data suggest circuit rewiring in Rlm1 and Sko1 CWI signaling [134]. Sko1 function has been extensively studied in *S. cerevisiae* and shown to be part of the MAP kinase high osmolarity glycerol, Hog, signaling pathway with a role in osmotic and oxidative stress responses [147]. The Hog pathway in *C. albicans* is associated with pathogenicity traits and it is involved in the control of both pathogenic and commensal state programs [148]. Sko1 function as the regulator of osmotic stress is conserved in *C. albicans* and it is phophorylated by the MAP kinase Hog1 following osmotic shock [133]. However, Sko1 regulates genes in *C. albicans* whose orthologues in *S. cerevisiae* are not involved in osmotic stress response, therefore showing circuitry rewiring [149]. Sko1 function in regulating the oxidative stress response is also conserved in *C. albicans* [150].

A *sko1*Δ/Δ mutant is hypersensitive to caspofungin, but not to Congo red, CFW, or SDS [148,150], suggesting Sko1 may not have such a global role in cell wall architecture as Rlm1 or Cas5. Microarray and RT-qPCR data demonstrated that Sko1 regulates 81 caspofungin-inducible genes and 26 of these genes are upregulated by Sko1 [133]. Several of the genes regulated by Sko1 have cell wall-associated function (*PGA13* and *CRH11*), and cell metabolism functions [133,134].

The upstream regulatory mechanisms controlling Sko1 expression in *C. albicans* are more complex than in *S. cerevisiae*, where the Hog pathway principally regulates Sko1 transcriptional activity. Caspofungin-induced Sko1 activity is independent of Hog pathway function [133]. RT-qPCR data demonstrated that caspofungin markedly induces *SKO1* transcription and this requires the glucose-partitioning PAS kinase, Psk1 [133], but Psk1 does not regulate transcription directly. Microarray data indicate that Rlm1 regulates *SKO1* expression under basal conditions [128]. Furthermore, caspofungin-induced *SKO1* expression is markedly reduced in *rlm1*Δ/Δ mutant but not in *cas5*Δ/Δ mutant. It is unknown if Psk1 binds directly to Rlm1 to regulate its activation of *SKO1* expression. However, a DNA-binding consensus has been identified in the Sko1 promoter sequence for regulating Sko1 inducible genes and also for autoactivation of *SKO1* [134].

The Hog pathway has not been well studied in other *Candida* species. However, in clinical strains of *C. auris*, Hog1 and Ssk1 have been shown to have variable activities, which suggested some sort of genetic flexibility with effects on cell wall function and stress adaptation [151]. A *C. auris ssk1*Δ/Δ *hog1*Δ/Δ mutant had altered tolerance to caspofungin and amphotericin B, with increased echinocandin susceptibility [151]. The mutant also had altered cell wall mannan content and altered hyper-resistance to cell wall stressors [151]. Targeting these two signaling components of the Hog pathway may provide options for an effective combination therapy or enhancement of echinocandin susceptibility.

Phosphotransferase regulator Ypd1 and phosphatase Ptp2 have been identified as the Sko1 targets following caspofungin treatment of *C. albicans* [134]. Both Ypd1 and Ptp2 are known to inhibit the Hog pathway, indicating that Sko1 blocks the Hog pathway following caspofungin treatment [152,153]. There is also cross communication between the Hog1 and Cek1 pathways under basal condition [154] and *C. albicans hog1*Δ/Δ mutants have constitutively higher levels of Cek1 phosphorylation [117].

The Cek1 pathway is involved in cell pathogenesis and participate in cell wall construction [155,156]. Cell surface signals that activate the Cek1 pathway are transmitted by membrane bound sensor: Sho1, Msb2, and Opy2 [154,155,156], and mediated through Cph1 and Tec1 [157,158]. Signals through the sensors trigger stimulus through Cst20 to the Ste11-Hst7-Cek1 MAPK cascade [119]. Deletion of any of these downstream elements as well as Cph1 does not affect filamentation [159]. Cek1 has also been shown to target another transcription factor, Ace2 to upregulate genes encoding protein *O*-mannosyltransferases in response to defective protein *N*- or *O*-glycosylation activities [160]. Cell surface proteins are post-translationally modified to maintain cell wall structure. Genes encoding components in the Cek1 pathway, *MSB2*, *CST20*, *HST7*, *CEK1*, and *ACE2* are Ace2 targets, indicating Ace2-mediated transcriptional upregulation of pathway genes under *N*-glycosylation stress [160].

In *C. albicans* and most other fungi, cell damage through the inhibition of β-1,3-glucans synthesis triggers compensatory chitin synthesis [101,114,161,162,163]. We have shown that Pkc, Hog, and Ca^2+^ signaling pathways co-ordinately regulate chitin synthesis in response to cell wall stress [72,110,163]. These pathways regulate *CHS* gene expression and chitin synthesis individually and in concert, leading to rearrangement of wall macromolecules in response to cell wall stresses [110]. A *lacZ* reporter gene was fused to the putative promoters of each of the *CHS* genes of *C. albicans* to monitor the expression of *CHS* genes when treated with cell wall perturbing agents such as CFW and showed that exogenous Ca^2+^, which induces the calcineurin pathway, activated all the *CHS* genes in a Crz1-dependent manner [110]. Crz1 is the downstream transcription factor in the Ca^2+^/calcineurin signaling pathway. Treating *C. albicans* cells with CFW, which activates the Pkc pathway, results in a three-fold increase in chitin content [101]. However, hyper-stimulation of *CHS* gene expression was observed when Pkc and Ca^2+^ pathways were simultaneously activated, and this resulted in increased chitin in the cell wall [110]. In *S. cerevisiae* and *C. albicans*, the Pkc and Hog MAP kinase cascades and the Ca^2+^/calcineurin pathway have been shown to regulate CWPs, such as Sed1, Pst1, Crh1, Cwp1, Ssr1, Yps1, Pir1, and Pir3, involved in cell wall remodeling activities [51,134,145,164,165,166,167].

The Ca^2+^/calcineurin signaling pathway is implicated in the activation of cell wall remodeling processes in response to damage to the cell wall [52,101] (Figure 1). The proposed model for Crz1 regulation in *C. albicans* is that the influx of Ca^2+^ activates calcineurin that then dephosphorylates and activates Crz1. The activated Crz1 enters the nucleus and binds to one or both Crz1 binding motifs in the promoter of target genes leading to their expression [168]. Crz1 has been shown to regulate the expression of 34 genes involved in cell wall biosynthesis in response to calcium stress and 12 of these genes encode proteins that are covalently bound to the cell wall: *CRH11*, *UTR2*, *PGA1*, *PGA6*, *PGA13*, *PGA23*, *PGA39*, *PGA52*, *PGA20*, *ECM331*, *PHR2*, *DFG5* [168]. Microarray and RNA sequencing data have reveal that Crz1 binds in vitro and in vivo to two identified motifs (calcineurin dependent response element, CDRE) in the promoter of some of the target genes [53,168] to induce their expression. The promoter of 79 genes regulated by Crz1 have two binding motifs for Crz1, while 104 Crz1-regulated genes have only one motif. Meanwhile, 36 Crz1 regulated genes have no discernible Crz1 binding motifs [168]. This suggests that the expression of Crz1 target genes is differentially regulated. It has been shown that Crz1 binds to two motifs in the promoter region of *UTR2* to induce expression in response to calcium stress [168].

*C. albicans* lacking calcineurin is markedly attenuated in virulence in a murine model of systemic candidiasis and cannot survive in the presence of cell membrane stressors [169,170,171]. *C. albicans* lacking Crz1, the major target of calcineurin is partially virulent in a murine model of systemic candidiasis, indicating the existence of other calcineurin targets that are important for virulence [168,172,173].

Another determinant of caspofungin sensitivity is the transcription factor, Cup9, which is required for normal caspofungin tolerance in hyphae alone and activates the expression of CWPs with cell wall function [174]. *C. albicans cup9*Δ/Δ mutant is hypersensitensive to caspofungin stress. RNA-seq data from *C. albicans cup9*Δ/Δ mutant with or without caspofungin demonstrated that Cup9 has a narrow rather than global effect in the cell wall damage response and activates proteins such as *PGA31* and *IFF11* with a known role in cell wall integrity [174].

Generally, these signaling pathways have not been studied in detail in other *Candida* species; however, in *C. glabrata*, 3 genes: *SLT2*, *YPK2*, and *YPK1*, whose protein products are involved in cell wall maintenance are associated with in vivo and in vitro echinocandins tolerance [175]. A *C. glabrata* strain lacking these three genes was susceptible to caspofungin treatment in a murine model of gastrointestinal candidiasis [175]. Furthermore, genes encoding ortholoques of kinases in the cell wall signaling pathway *SLT2*, *MKK1*, *BEM2*, and *SW14* were identified in *C. glabrata* as well as genes encoding ortholoques of calcineurin pathway membrane components: *CCH1* and *MID1* [146]. Mutants lacking any of these genes were hypersensitive to caspofungin [146]. Deletion of genes representing all stages of CWI pathway from surface sensing to transcription regulation resulted in various degrees of susceptibility to caspofungin and cell wall degrading enzymes [146]. Although more studies are required to understand caspofungin-induced cell wall stress responses in other *Candida* species, available data suggest similarity in signaling pathways, the response strategies deployed, and the wall proteins involved in maintaining cell wall integrity.

## 4. Cell Wall Remodeling in Response to Thermal Stress

**Figure 2:**
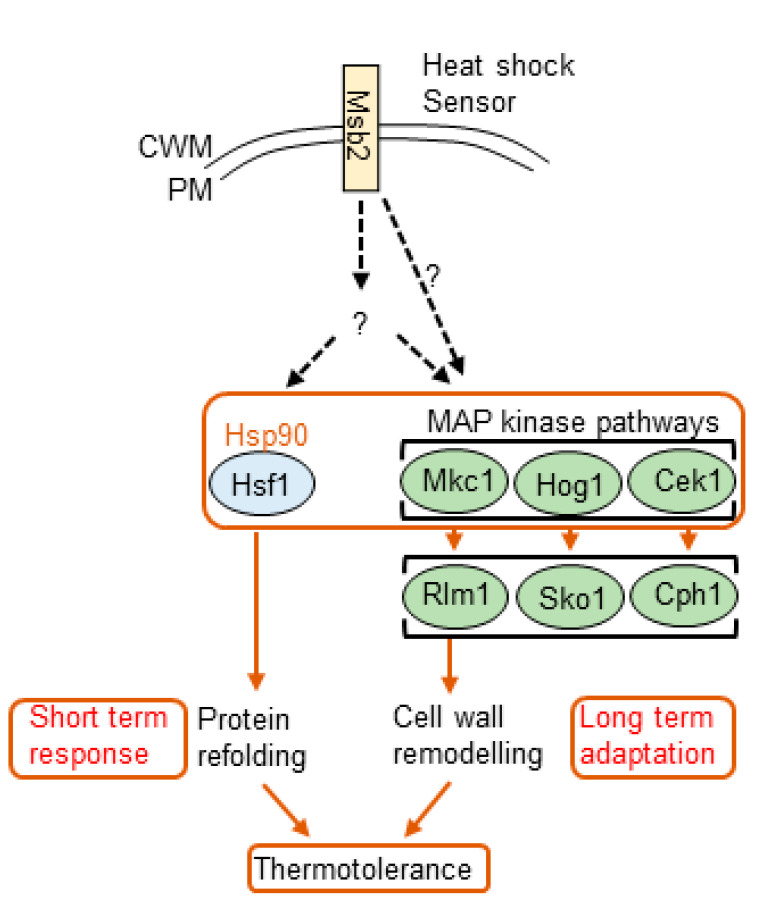
Hsp90 acts as a biological transistor, modulating Hsf1 and the MAPKs transcription factors in response to thermal fluctuations. Msb2 plays a vital role in thermotolerance in *C. albicans*. The protein transmits heat shock signals through unknown mechanisms that induce downstream targets such as the Pkc pathways in response to high temperatures. Hsf1 activation is required for thermotolerance. The MAP kinase signaling pathways are also required to promote thermotolerance through remodeling the cell wall [117,185]. Because Hsp90 coordinates much of this activity, Hsf1, Hog1, Mkc1, and Cek1 are all thought to be Hsp90 client proteins [177,181]. Fluctuations in ambient temperature affect interactions between Hsp90 and Hsf1, and probably affect Hsp90 interactions with the MAP kinase transcription factors [181], thus modulating the role of the signaling pathways and thermal adaptation outcome. Temperature upshifts activate Hsf1, which induces the expression of protein chaperones (HSPs), including Hsp90, which promotes shorter term thermal adaptation. It is thought that Hsp90 then down-regulates Hsf1 and modulates MAP kinase signaling, to alter cell wall architecture, which leads to long term thermotolerance in *C. albicans*. Adapted from [177]. Broken lines indicate unconfirmed regulatory mechanisms.

The fungal response to heat shock has been well characterized [176,177,178]. Temperature stress signals are thought to be sensed by signaling mucins. Signaling mucins are transmembrane glycoproteins that receive and transmit surface signals to signaling pathways (Figure 2). Signaling mucin, Msb2 is known to regulate environmental stress, cell wall biogenesis, and the Cek1 and Pkc pathways in most fungi [178,179]. Msb2 is a global regulator of temperature stress in *C. albicans* [113]. Msb2 is required for fungal survival and hyphae formation at 42 °C. Msb2 also regulates temperature-dependent activation of genes involved in MAP kinase and unfolded protein response pathways (Figure 2) [113].

Generally, the temperature stress response is controlled by an essential protein, the heat shock transcription factor, Hsf1, which is phosphorylated upon sudden temperature rise [180]. Following temperature rise from 30 to 42 °C, Hsf1 is phosphorylated rapidly within 60 s and upon adaptation, downregulated [181]. Under normal growth conditions, Hsf1 binds as a trimer to heat shock elements (HSEs) in the promoters of target heat shock protein (*HSP*) genes [182]. When *S. cerevisiae* or *C. albicans* cells experience an acute heat shock, Hsf1 is hyper-phosphorylated and activated, resulting in the transcriptional induction of the target *HSP* genes, thus stimulating cellular adaptation to the thermal insult [183]. Most heat shock proteins, Hsp, are molecular chaperones that promote client proteins folding, assembly, or cellular localization. They also often target unfolded or damaged proteins for degradation [184]. In *C. albicans*, Hsf1 interacts with Hsp such as Hsp90 under steady-state conditions, and upon thermal shocks, this interaction is strengthened, suggesting existence of a Hsf1-Hsp90 autoregulatory circuit [177]. Hsp90 is localized to the nucleus during elevated temperatures. It is possible that the Hsf1-Hsp90 regulon is critical for the maintenance of thermal homeostasis, not merely for adaptation to acute heat shocks. This suggests that the Hsf1-Hsp90 interaction is important for regulation of short-term responses to heat shock (Figure 2).

Cell wall integrity is compromised at elevated temperatures. Temperature affects cell wall polysaccharide composition and the incorporation levels of covalently anchored proteins [186]. Yeasts cells are thought to adapt to heat stress in the longer term by activating the Hog1, Mkc1, and Cek1 MAP kinase pathways, which contribute to thermotolerance [177,186] (Figure 2). These MAP kinase pathways, even though they contribute to thermal adaptation in the longer term through cell wall remodeling, are not essential for Hsf1 activation. Genetic depletion of Hsp90 affects cell wall remodeling activities, suggesting that Hog1, Mkc1, and Cek1 may be client proteins of Hsp90. Hsp90 is thought to be able to integrate both the short term and longer-term molecular responses that underpin thermotolerance [177] (Figure 2).

In *S. cerevisiae*, MAP kinase pathways have been shown to contribute to thermotolerance [132,187], through localization of Chs3 to the plasma membrane in response to heat shock [129]. Each of these MAP kinase pathways is known to contribute to cell wall remodeling and mutations that interfere with cell wall synthesis increase sensitivity of *C. albicans* to elevated temperatures. For example, the deletion of certain protein mannosyltransferases of the PMT family, or the inactivation of *OCH1* can increase susceptibility to temperature [188,189]. Furthermore, deletion of *SSR1* causes elevated susceptibility to temperatures [60].

In a study, thermal upshift was shown to cause reduced secretion of chitinases and have a huge impact on cell wall *N* mannan composition [190]. Analysis of the cell wall phospholipomannan moiety revealed reduction in *N* mannan composition of β-1,2-mannose [190]. *C. albicans* is more susceptible to cell wall stressors when grown at 42 °C [186]. Coping with this thermal stress leads to increased phosphorylation of Mkc1, which mediates activation of the CWI pathways. Consequently, the levels of Sap9, the chitin transglycosylases Crh11 and Utr2, and the cell wall maintenance protein, Ecm33, increased, and cells reinforce their walls with chitin through increased chitin synthesis and reduced chitin degradation [186]. Ecm33 is required for growth at high temperatures and *S. cerevisiae* and *C. albicans* *ecm33*∆/∆ disruptant strains exhibit a temperature sensitive growth defect [191,192].

The Mkc1, Hog1, and Cek1 signaling pathways and associated cell wall remodeling mannoproteins have been proposed to promote longer term thermotolerance through the maintenance of a robust cell wall (Figure 2).

## 5. Echinocandin-Induced Cell Wall Remodeling in Yeast

β-1,3-glucan is a hallmark component of most yeast cell walls and is synthesized by β-1,3-glucan synthase. The protein has an integral membrane catalytic subunit, Fks [193]. *C. albicans* has three *FKS* genes, but the main activity is from the *FKS1* gene product, Fks1. Fks1 is essential and found in association with the regulatory subunit, Rho1 GTPase [194]. Rho1 is required to activate Fks1 for β-1,3-glucan synthesis (Figure 1). Echinocandins non-competitively inhibit β-1,3-glucan synthesis by inhibiting the catalytic function of Fks1, leading to a weak cell wall [195]. Echinocandins are fungicidal against *Candida* species and resistance to the drug has been predominantly associated with point mutations in the *FKS1* gene. However, most yeast have been shown to withstand caspofungin treatment, becoming more tolerant to the drug both in vivo and in vitro by inducing the upregulation of chitin synthesis, the second wall structural polysaccharide [162,163].

Chitin is synthesized by chitin synthase enzymes and *C. albicans* has four chitin synthase proteins comprising of *Chs1*, *Chs2*, *Chs3*, and *Chs8*. Elevated cell wall chitin is a cell wall rescue mechanism shown to be orchestrated by the CWI pathways [101,110,196]. Pkc, Hog, and Ca^2+^ signaling pathways have been shown to control the expression of *CHS2* and *CHS8* through binding motifs in their promoter sequences [131]. Hyper-stimulation of *CHS* gene expression was observed when the three signaling pathways were activated at the same time and this leads to elevated cell wall chitin content [110]. Cell wall mutants with higher basal chitin contents are also less susceptible to caspofungin [60,197]. Chitin synthase proteins can also synthesize alternative septa that restore *C. albicans* capacity to bud during cell wall stress [198].

Genome wide studies have been carried out to study the response of fungal cells to echinocandin drugs treatment and to identify genes whose upregulation is required for adaptive growth in the presence of sub-MIC concentrations of echinocandins. DNA microarrays studies identified genes that are activated in *S. cerevisiae* and *C. albicans* when they are challenged with sub-MIC concentrations of caspofungin [54]. The induced genes include those genes that are typically upregulated following the activation of the Pkc pathway. In *C. albicans* and *S. cerevisiae*, some of the Pkc pathway signature genes: *CRH11*/*CRH1*, *ECM331*/*PST1*, *DFG5*, encode GPI anchored cell surface proteins that have been implicated in cell wall biogenesis or repair [51,55,199,200,201,202]. Pga31, a predicted GPI anchored wall protein, is upregulated during caspofungin stress, and *pga31*Δ/Δ mutants have thinner cell walls, reduced chitin content, and are hypersensitive to caspofungin [60]. Cas5 regulates the expression of some CWPs in response to caspofungin, including Crh11, Ecm331, Pga13, and Pga23 [54]. Pga13 plays a role in cell wall architecture [203] and may be required for cell wall repair.

The phosphorylated form of the Pkc pathway component Mkc1/Slt2 and phosphorylated form of Cek1 have been detected in *S. cerevisiae* and *C. albicans* [155] following caspofungin challenge. Furthermore, *C. albicans mkc1*Δ/Δ mutant is hypersensitive to caspofungin [101]. This suggests that the Pkc pathway is a major signaling pathway for triggering cell wall macromolecule rearrangement in response to caspofungin stress in *S. cerevisiae* and *C. albicans* [101,196].

## 6. Cell Wall Remodeling and Protein Abundance

In an analysis of the cell wall proteome of *C. albicans* growing on minimal medium without stress using liquid chromatography-mass spectrometry, LC-MS revealed 21 covalently bound CWPs. Out of the 21 CWPs identified, 19 had predicted GPI anchor sequence with cell wall associated function [204]. In other studies, the proteomics technique was used to study the impact of carbon source on the *C. albicans* cell wall proteome and secretome when cells were grown in minimal medium containing 2% glucose, lactate, or glucose plus lactate [73,205]. The results revealed higher amounts of predicted GPI anchored CWPs with functions in cell wall biogenesis/integrity in the secretomes and proteomes. Major differences were seen in the profiles of secreted and CWPs in lactate and glucose-grown *C. albicans* cells. Many of the differences suggested that specific cellular processes associated with the cell surface such as cell wall remodeling, adherence, and biofilm formation, may be affected by the change in carbon source [73]. The secretome and proteome of lactate grown cells had increased levels of proteins involved in the remodeling of β-glucan [73]. Lactate grown cells were more adherent, and consequently, more virulent in in vivo models of systemic candidiasis and vaginitis, and display increased resistance to caspofungin as well as other stressors [111]. Lactate signaling regulates glucan masking and modulates the immune response [206]. Furthermore, elevated stress resistance did not correlate with increased activation of the CWI pathways, thus the observed phenotypes may be due to the alteration in the architecture as well as the biochemical and biophysical properties of the cell wall [111]. However, Hog1 or Mkc1 signaling pathways mediate expression of CWPs that promote cell wall elasticity required for adaptation to hyperosmotic stress [52]. Interestingly, alterations in the cell wall in response to different media or carbon sources have been shown to involve changes in the molecular weight of mannoproteins [207]. Mannoproteins from *C. albicans* cultivated on blood or serum have increased molecular weight, when compared with mannoproteins from cells grown on YPD at 30 and 37 °C [207].

A microarray study using DAY185 *C. albicans* strain with or without caspofungin treatment identified 216 caspofungin-inducible genes with an expression change of at least two-fold following 1-h caspofungin treatment [54]. A core set of 34 caspofungin stress inducible genes included genes that are known to be involved in cell wall remodeling such as *PGA13*, *CRH11*, and *PHR1* [54]. In addition, *C. albicans* grown in vagina-simulative medium, aerated with a gas mixture reflecting the gas composition in the vaginal environment had five CWPs [Als3, Hwp1, Sim1, Tos1, Utr2) in the wall that were absent in the YPD grown control [38]. However, O_2_ restriction led to higher levels of the non-GPI protein Pir1, β-1,3-glucan cross-linking protein, and of the GPI anchor protein, Hwp1, an adhesion protein [38].

Environmental pH has also been shown to greatly alter the fungal cell wall proteome. Klis lab used a system that mimics mucosal surfaces to investigate the influence of host pH on *C. albicans* cell wall proteome [208]. At pH 4.0, yeast cells and pseudohyphae were predominantly seen while at pH 7.0, hyphal growth was mainly seen. Relative quantitation of ^15^N-labelled CWPs using ESI-FT-MS revealed the identity of 21 covalently linked CWPs, most of which are GPI anchored, excluding Tos1, Mp65, and Pir1. At pH 7.0, Als1, Als3, Hyr1, Phr1, Rbt1, Sod5, and Tos1 were identified, while only the transglycosidase, Phr2 was found at pH 4.0. Furthermore, at pH 4.0, 12 out of the 21 CWPs were overexpressed, whereas at pH 7.0, 9 proteins were overexpressed. The proteome of the *C. albicans* cell wall is constantly reshuffled to enable cells to adapt to prevailing environmental conditions. The consequences of not adapting to that changing environment is cell death. This is why the cell wall, and its components, are attractive targets for developing more effective diagnostics and therapeutics.

## 7. Perspective

The covalently bound CWPs in the protein coat are indispensable for the survival of *C. albicans* in the environment and during infection. They also play a major role in the development of biofilms and are regulated by signaling pathways that help remodel the cell wall during stress. However, our knowledge of their structure, which may influence their function regarding structure-function relation is limited and our understanding of their exact function in many cases is still poor. This calls for a continued functional analysis of fungal CWPs. The regulatory mechanisms associated with the construction of the cell wall protein coat are not well understood. The precise mechanism of coupling these proteins to cell wall and their method of interaction with wall polysaccharide and other proteins in the cell wall, which may affect their localization and hence their function, are still not clear. Understanding the function and regulatory mechanisms of these CWPs will ultimately inform our knowledge of fungal pathogenesis and host-pathogen interactions.

CWPs have carbohydrate-binding motifs and may thus be involved in cell wall synthesis and remodeling, in biofilm formation, or even in the interaction with host cell receptors or other environmental signals. Most importantly, our knowledge of the exact roles CWPs play in CWI pathways, their downstream signaling activities, and the extent of their involvement in the cross interactions between the pathways during cell wall stress is relatively unexploited. The cell wall proteome can change significantly in response to specific environmental stress, including during infection. The fungal cell wall proteome changes associated with infection conditions need more extensive studies, as the cell wall in vivo is likely to be very different to the wall generated under laboratory growth conditions. Finally, the relative and absolute quantitation of CWPs under host-related conditions and an extensive understanding of their exact structure and functions will be vital in identifying the most suitable diagnostic, therapeutic, and vaccine candidates.

## Figures and Tables

**Table 1:** Characteristics of specific *Candida albicans* surface proteins.

Protein/Family	Features and Functions	Regulation
**GPI modified CWPs** **Adhesins, invasins** **Als family** (Als1-7, 9)		
	N-terminal c. 300-residue Ig domain, bind variety of substrates [27,28]; high (Als1, Als2), intermediate (Als4, Als9), and low (Als5-7) levels of gene expression [27,29,30,31]. Als3 is expressed uniformly all over hyphae [32]. Als1 and Als3 N terminal sequences are used as vaccine antigen [7]. Als1 and Als3 contribute to biofilm formation, and Als3 functions as an invasin, and as a ferritin receptor [27,30,31,33].	Als proteins are differentially expressed, *ALS1*, *ALS3*, and *HWP1* are under the positive regulatory control of Bcr1 [34,35,36]. Tup1 (repressor of filamentation] and Ahr1 are required for full expression of *ALS3* [37].
**Hwp1, Hwp2, Eap1, Ihd1, and Hyr1**	Hwp1 level is induced by oxygen and iron restriction [38]. N terminal is recognised as substrate for epithelial transglutaminases [39]. Hwp1 facilitates cell to cell interaction important in biofilm development [33]. N terminal 14-mer peptide and recombinant N terminal fragment are used in vaccine and diagnostic development, respectively [40,41]. Hwp2 has sequence identity with Hwp1 and can function in adhesion and invasion; it is also involved in oxidative stress tolerance and protein aggregation [42,43]. Hwp1, Hwp2, Eap1, and Ihd1 contribute to initial cell attachment and adhesion maintenance during biofilm formation [44]. N terminal of Hyr1 has been used in vaccines and diagnostics development [5,45].	
**Carbohydrate active enzymes**		
**1,3-β-Glucan processing Phr1-3, Pga4, and Pga5**	N terminal glycoside hydrolase (GH) 72 domain; play a role in cell wall construction (β-1,3-glucan modification); incorporated at acidic pH (Phr2) and neutral/alkaline pH (Phr1) [45,46]. Pga4 is transcribed independent of pH, and Phr3 and Pga5 have low expression levels [47]. Pga4 is serum- and host infection-inducible [48].	*PHR1* and *PHR2* are differentially regulated by extracellular pH [49].
**Chitin-glucan cross-linkers** **Chr family**	N terminal GH16 domain; involved in cell wall organization and integrity; cross-linking β-1,3-glucan and chitin; involved in protoplast regeneration [50,51]. Control cell wall elasticity in osmotic resistance [52].	*UTR2* expression is regulated by calcineurin and Crz1 [53]. *CRH11* is subject to caspofungin-induced Cas5 regulation [54]
**Others**		
**Dfg5 and Dcw1**	Putative glycosyltransferase enzyme activity; involved in the incorporation of GPI anchored proteins into the cell wall [55,56]. Dfg5 and Dcw1 are involved in hyphal morphogenesis and biofilm formation; Dfg5 is required for growth; Dcw1 is required for cell wall integrity response; Dfg5 has synthetic lethality with Dcw1 [56].	*DFG5* has been shown to be regulated by Rlm1 in *S. cerevisaie*, but not in *C. albicans* [54].
**Pga31-like** (Pga29-31)	Enriched in pathogenic fungi [57]. Pga31 has predicted transmembrane domain and with Pga30 they have three conserved cysteine residues (http://www.candidagenome.org/ (accessed on 12 July 2021)). Pga29 and Pga31 are echinocandin induced; Pga29 is required for normal cell surface property [58]. Pga31 is induced during protoplast regeneration [59] and may be involved in cell wall chitin synthesis during remodelling in response to stress [60].	*PGA31* is upregulated by the Pkc pathway [60].
**Sod4 and Sod5**	Superoxide dismutase; contribute to combating oxidative stress by clearing reactive oxygen species [61].	Rim101 is required for induction of *SOD5* under certain conditions, and Efg1 is required specifically for serum-modulated expression [62].
**Sap9 and Sap10**	Yapsin-like proteins are mainly found in the cell membrane (Sap9) and cell wall (Sap10); required for full cell wall integrity [63].	
**Pga59**	Cell wall localised [64]; abundant in the cell wall protein coat; mature protein consists of three cysteine residues and cross-links cell wall proteins through disulphide bridges [64].	
**Rbt5,Pga10,** **andPga7**	N terminal CFEM domain; cell membrane (Pga7 and Rbt5) localised; loss of function results in fragile biofilms (Pga10 and Rbt5) [65,66]; function as haeme receptors and involved in haeme-iron utilization [67,68]. Rbt5 levels increase following iron and oxygen restriction [38].	The proteins have been shown to be expressed during yeast to hyphae switch and thus are regulated by Tup1 [66].
**Non-GPI modified CWPs**		
**Pir1**	C terminal conserved four cysteine pattern and seven repeats; predicted to cross-link β-1,3-glucan chains [69]; protein levels increase in hypoxic conditions [38].	
**Mp65**	C terminal GH17z domain; present in fibrillar material with putative transglycosylase activity; potential vaccine candidate [70].	
